# Stroke survivors’ views and experiences on impact of visual impairment

**DOI:** 10.1002/brb3.778

**Published:** 2017-08-13

**Authors:** Fiona J. Rowe

**Affiliations:** ^1^ Department of Health Services Research University of Liverpool Liverpool UK

**Keywords:** adaptation, biographical interviews, daily living, formal care, impact, information, stroke, symptoms, visual impairment

## Abstract

**Objectives:**

We sought to determine stroke survivors’ views on impact of stroke‐related visual impairment to quality of life.

**Materials and Methods:**

Stroke survivors with visual impairment, more than 1 year post stroke onset, were recruited. Semistructured biographical narrative interviews were audio‐recorded and transcribed verbatim. A thematic approach to analysis of the qualitative data was adopted. Transcripts were systematically coded using NVivo10 software.

**Results:**

Thirty‐five stroke survivors were interviewed across the UK: 16 females, 19 males; aged 20–75 years at stroke onset. Five qualitative themes emerged: “Formal care,” “Symptoms and self,” “Adaptations,” “Daily life,” and “Information.” Where visual problems existed, they were often not immediately recognized as part of the stroke syndrome and attributed to other causes such as migraine. Many participants did not receive early vision assessment or treatment for their visual problems. Visual problems included visual field loss, double vision, and perceptual problems. Impact of visual problems included loss in confidence, being a burden to others, increased collisions/accidents, and fear of falling. They made many self‐identified adaptations to compensate for visual problems: magnifiers, large print, increased lighting, use of white sticks. There was a consistent lack of support and provision of information about visual problems.

**Conclusions:**

Poststroke visual impairment causes considerable impact to daily life which could be substantially improved by simple measures including early formal visual assessment, management and advice on adaptive strategies and self‐management options. Improved education about poststroke visual impairment for the public and clinicians could aid earlier diagnosis of visual impairments.

## 1 INTRODUCTION

Approximately 60% of stroke survivors have visual impairment (Hepworth et al., 2015) which typically relates to impaired central or peripheral vision, eye movement abnormalities, or visual perceptual defects (Rowe & VIS Group, 2009a). Resultant symptoms can include blurred or altered vision, double or jumbled vision, loss of visual field, reading difficulty, inability to recognize familiar objects or people and glare (Rowe & VIS Group, 2013). Poststroke visual impairment is an under researched area and is cited as a research priority in two national surveys by the James Lind Alliance (Life after Stroke [Pollock, St George, Fenton, & Firkins, 2014]; Sight loss and vision [Rowe et al., 2014]) along with being a reported unmet need for stroke survivors (Coleman, 2010; Rothwell, Boaden, Bamford, & Tyrrell, 2012).

Poststroke visual impairment leads to impact on daily life. Studies reporting impact through collated responses from quality of life questionnaires outline the impact including loss of confidence, fear in independent mobility/navigation, inability to drive, inability to read, social isolation and depression (Hepworth & Rowe, 2016). Responses from patient‐reported outcome measures are directed by the items included in these questionnaires. There is currently a paucity of evidence of the “lived” experiences, views, or needs of stroke survivors with visual impairment. A greater understanding of their needs could provide information to guide, develop, and improve the organization and delivery of care across acute and community services. The purpose of this study was to ask stroke survivors directly about their experiences and views of having visual impairment.

## 2 METHODS

### 2.1 Study design

This study was undertaken in accordance with the Tenets of Helsinki. Ethical approval was obtained from the Institute research ethics committee and written, informed consent was obtained from all participants.

In this prospective study, biographical narrative interviews were carried out with 35 adult stroke survivors, conducted by the author. In‐depth, face‐to‐face interviews (typically 1–2 hr duration) commenced with a preconstructed single narrative question. This was followed with additional narratives relating to the sequence of topics raised and then followed by nonnarrative questions to explore the topics further. During the interview, stroke survivors were asked about what their visual symptoms or difficulties were, how these impacted on their activities of daily living and quality of life, what assessments and treatments they had (if any), and what they wish to see happen in future patient care.

### 2.2 Recruitment

We contacted patient and public forums such as Connect, Different Strokes, Speakability, Stroke club consultation group, the Stroke Association, North West Stroke Research Network consumer reference panel, Royal National Institute for the Blind, North West People In Research forum, and local patient involvement groups. These groups circulated the recruitment announcement to stroke survivors and carers through network emails and social media interactions such as Facebook and Twitter.

Stroke survivors interested in the project contacted the chief investigator (FR) and were sent a participant information sheet with further details of the project. This was followed up by a telephone call to the stroke survivor by the author to discuss the project and any queries arising from the participant information sheet. If the stroke survivor wished to proceed, an appointment was made for interview. All but one interview was conducted in patients’ homes with one interview taking place in a local community center. At the interview appointment, the stroke survivor was given the opportunity to ask any further queries about the project and, when satisfied with the information, informed written consent was obtained in accordance with Institutional ethical approval requirements.

### 2.3 Participants

The target population was long‐term (>1 year since stroke onset) stroke survivors with visual problems that had occurred due to the stroke. Inclusion criteria included stroke survivors aged 18 years of age or older who had suffered visual impairment following their stroke and with the ability to agree to take part in an interview and able to provide informed written consent. Exclusion criteria included stroke survivors less than 18 years old and those with severe cognitive or communication impairment preventing interview. In the design of this study, we set an initial target sample size at 35 based on a medium‐size estimate (Baker & Edwards, 2012). However, at all times the intention was to continue increasing the number of interviews until saturation was achieved, even if this would take the sample size higher. In practice, a higher sample size was not required.

### 2.4 Analysis

Interviews were audio‐recorded, transcribed verbatim and all identifying features removed. Qualitative data analysis was undertaken as an ongoing iterative process (Ritchie & Lewis, 2007). All transcripts were systematically coded using the NVivo10 qualitative software package.

Each transcript was read while listening to the audio file. This allowed checks of transcript accuracy and provided an overview for each transcript on issues raised by the participant. Transcripts were coded by sentence or section and the code descriptors were derived directly from the text. Separate independent analysis and coding was undertaken for five transcripts (>10%) for cross‐check capture of codes. The two sets of codes were compared to identify variation, agreement, and code descriptions assigned to text. This formed the development of a detailed coding scheme which was used to code all transcripts.

A thematic approach to analysis of the qualitative data was adopted. Codes were grouped for similar content and these groups defined the key emerging themes (Strauss & Corbin, 1990). A modified grounded theory approach was undertaken in which themes were revised iteratively as further interviews and analysis progressed.

All participants were sent a copy of their transcript for their feedback.

Descriptive quantitative statistics were used to report the number of participants experiencing types of visual problems, burden, and for reporting participant demographics and recurrent issues.

## 3 RESULTS

### 3.1 Interviewee demographics

**Figure 1:**
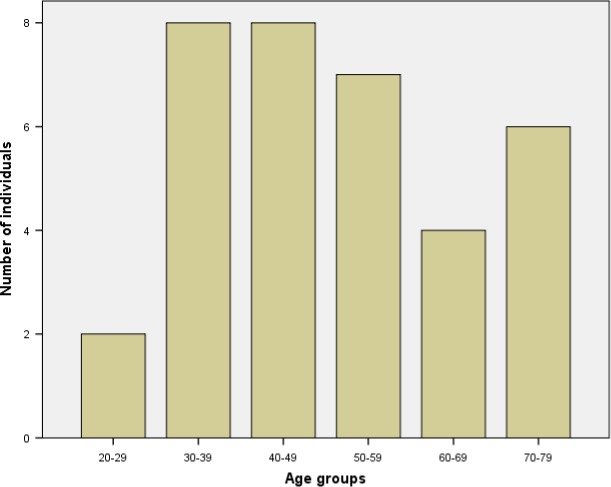
Participant age groups are per decade from the age of 20 through to 79 years of age

**Figure 2:**
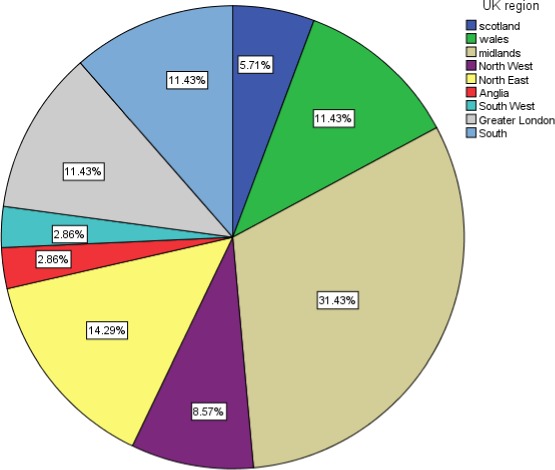
Geographical location is shown in the UK for participant homes

**Figure 3:**
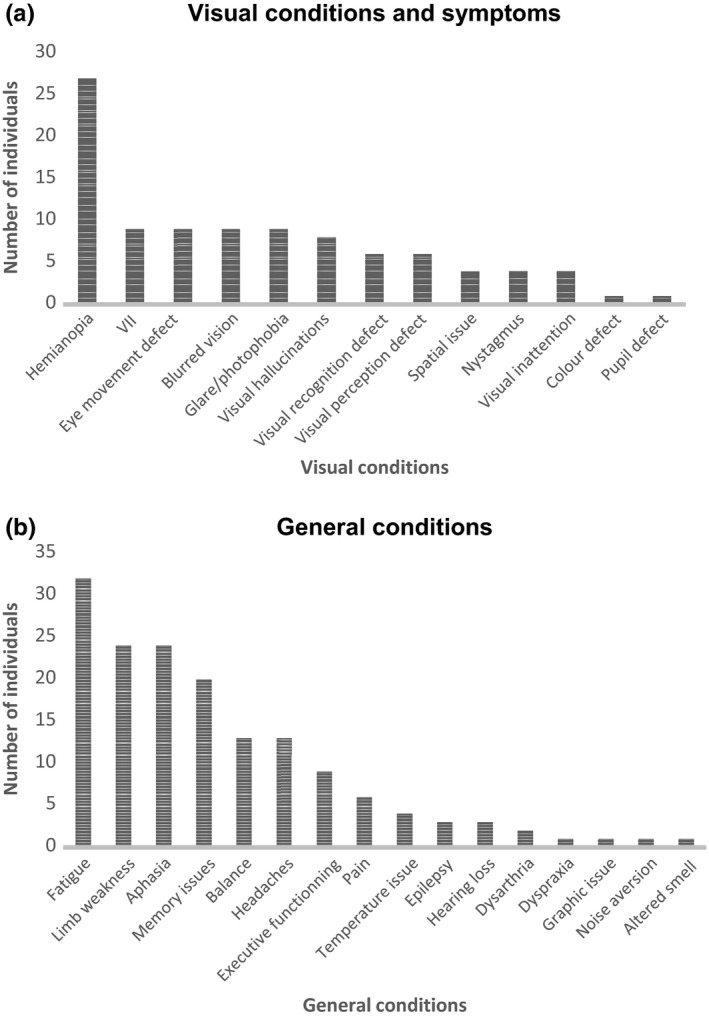
Visual conditions and symptoms reported by participants are shown in a. General conditions and disabilities documented for participants are shown in b

Thirty‐five stroke survivors were interviewed who had ischemic (*n* = 24) or hemorrhagic (*n* = 11) strokes. There were 16 females and 19 males aged from 20 to 75 years (Figure 1) at stroke onset. Interviews were conducted in England, Wales, and Scotland (Figure 2). One individual was of Asian ethnicity, one Black, and the remainder were Whites. The mean duration of time from onset of stroke to time of interview was 5.7 years (*SD* = 5: range = 1–22 years). Eight participants reported just one visual condition: four with homonymous hemianopia, two with eye movement defects, one with VIIth nerve palsy, and one with visual perceptual issues. The remaining participants had multiple visual conditions of which hemianopia was most common followed by eye movement defects, blurred vision, glare/photophobia, and visual hallucinations (Figure 3a). It was not possible to obtain reliable details of referral patterns for all participants as there was confusion of high‐street optometry services, community eye services, hospital eye departments, and vision services provided within stroke units. It was also not possible to provide specific details of the extent of visual impairment for each participant. Given the nature of this study design (interviews in participants’ homes), we did not have access to hospital notes to obtain medical details of stroke and resultant disabilities.

**Figure 4:**
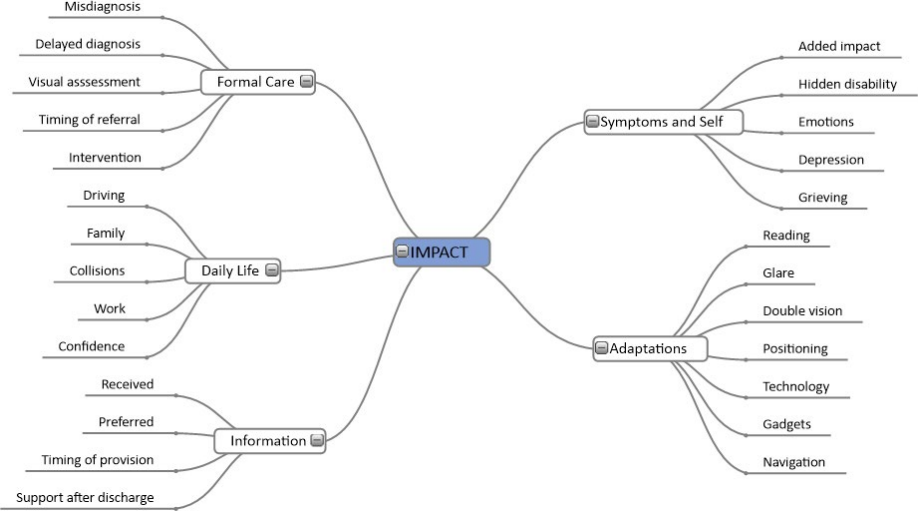
An outline of the five emergent themes arising from analysis of interviews alongside their individual code descriptors

Five themes emerged (Figure 4).

### 3.2 Formal care

Nineteen participants reported issues with misdiagnosis because of having visual impairment along with delays in their diagnosis of stroke. The stroke was diagnosed immediately in both participants in their 70s. There was a delayed diagnosis of >3 days in the younger participants.

Having visual impairment as the primary presenting symptom, particularly for younger participants, produced a variety of possible diagnoses such as migraine, meningitis, brain tumor, detached retina, multiple sclerosis, and Bell's palsy (Table 1A).

**Table 1:** Participant quotes

Formal care
**A: ** *he said erm right well I can't find anything wrong with your eyes it must be your brain and a couple of years before that I'd had erm my auntie had passed away from a brain tumour er so my mind just went right so I said to him so what could it be and he said well you've either got a tumour in your occipital lobe or a tumour on your optic nerve or I don't know what and I said so you think I've got cancer then and he said well we'll just wait and see* **B: ** *then this junior doctor came to see me and she sort of took me into a cubicle and she was like ok erm I, I, I can't really remember what she was asking me but she asked me if I had had a migraine before and I said no, and she said the symptoms are very erm similar to, to a migraine I said well it's been a couple of hours now and she said well you know it can last for days migraines* **C: ** *I mean they, they did say we, that you hadn't had a stroke because they said well you can stand up and stand on one leg and, so they said it's not a stroke, at that point* **D: ** *so he said right, he said well, it's, this was on the Thursday, erm, and then it was bank holiday day, Good Friday, the next day, so he said cause it's bank holiday, he said wait til Tuesday, he said, after the weekend, he said if you're still not right, go to the hospital, cause they've got a walk in eye clinic* **E: ** *February, March time and I remember I had to wait so long for an appointment and I think they offered me appointment in December I think that's how long it was, I think it was nearly 11 months* **F: ** *it was quite shocking to see the amount of it that was lost but in another way it made sense because I knew things weren't working right but because it then just made sense as to why these things were happening erm because I think otherwise it was just really difficult to know* **G: ** *you really need to get a really good assessment of all the bits how you are what's happening for you because I think otherwise you don't, you don't know what is wrong* **H: ** *he says oh there's nothing I can do for you, and he said erm, it's the stroke that's done it, and it was very matter of fact, and there was no feeling or anything behind it, and er, it was just one of those things, and it was like you've got it, get on with it, there's nothing I can do for you, and really that was it, erm, with regard to, to my experience about my vision*
Impact of symptoms
**I: ** *so yeah probably as that tiredness receded and you know life started to go back well not to normal but into it's new tangent it was then that the vision problem became more and more apparent* **J: ** *so that they (clinicians) would stand where I couldn't see them, or, or stuff would be put down on the, on my blind side where I didn't know was there, yeah, and sometimes I didn't even realise my erm, meal was there, you know, if you'd been dozing and you're waking up and you think, and the table has been moved round to the other side, and you think, and then suddenly you realise that something's sitting there, and you didn't know it was there* **K: ** *and again, that was only because physically, er, that, that's to look at me, I looked OK, but nobody was aware of what else was going on inside, and it felt as though they were totally oblivious to what I was going through, erm, because they couldn't see the cognitive side, they couldn't see the vision problem, erm, and as I say, if I had a paralysis, I actually think I'd have been treated differently* **L: ** *no in fact I'm really, really hot on wiping that word away because I don't think they are hidden I just think they are not obvious and if people don't understand or wish to understand or haven't the time or the understanding to look properly* **M: ** *with my eye, cause I can't see out this side, erm, it's, it makes me feel a little bit more vulnerable, you know when I'm walking and there's someone behind me* **N: ** *it has the emotional impact on you then because you feel as if you're a burden to everybody else, cause you've caused another mess,* **O: ** *the doctor asked me if I felt depressed, I said no I don't, I don't feel depressed at all I just feel a bit fed up now then and tired* **P: ** *that's a grieving process you're grieving for …. not just the loss of your vision but your old life*
Self‐made adaptations
**Q: ** *I've got a little magnifier that like, you know, makes it a bit bigger for me and makes it easier, and my husband bought me an iPad* **R: ** *because I'd lose my place, I used to use a ruler, and my thumb at the end* **S: ** *with the advent of sat nav and Google maps now when I go anywhere I have a sat nav in the car I always look at the destination I'm going to on Google maps and I familiarise myself with the locality ‘cause I still have a very good visual memory* **T: ** *erm, my jar openers and my tin opener, and my potato peeler, they got me those electronic ones* **U: ** *if I go somewhere and it's lots of people around, er, very busy, and I'm not sure of the area, and I know that something, I'll do something, or something like that, then I'd take it you know and use it, erm, because that, having the, the visual in, with the cognitive part, cause I do get confused at things, and, and if there's lots of people a, it's very difficult for me to take a lot of stuff in, and that's when things happen* **V: ** *I felt, I tended to put my hand out when I went to a wall just to steady myself, really, erm, and guide myself to a certain, to make sure I was walking far enough out so as not to hit a door post* **W: ** *I have learnt myself how to scan with my eyes all the time* **X: ** *kids were really good, they, they'd say, you know, we're on your left, you know, and they were good,* **Y: ** *yeah, closing one eye, er, was one thing … . I was so housebound that there was erm, I, I would tilt my head an awful lot to the left, cause that would make me feel good*
Daily life
**Z: ** *but at home, erm, that's when things really sort of came into their own in a way that I'd erm, start falling over things, I'd fall over the coffee table, I'd walk straight into the door, erm, I would er, I was covered in bruises, because I kept knocking into things, erm, I'd trip over the dog, I'd kick the dog, I couldn't see it, you know, erm, make, making in the kitchen I was a nightmare, erm, with boiling water and stuff I'd be filling something and I couldn't, I didn't see the cup* **Ai: ** *if you go into town, erm, and you bump into people, and, and they look at you sometimes as if you're either drunk or erm, you know, you're just not looking where you are going* **Bi: ** *since you have had this stroke erm, mainly it's because you said you can't see but a lot of it is to do with the fact that you haven't got the confidence to go out* **Ci: ** *I was followed everywhere, erm, it, it didn't matter what I did, if I went up to the, I'll do it, I'll get it, do you want, I'll get it, you know, erm, so yeah, they were scared, that something else was going to happen, erm, they wouldn't go out, erm, and they were frightened, oh no we'll just stay in, and it got to a stage that I had to say, OK, this is it, stop now, you're going to have to start, go out*
**Di: ** *if I go to a busy shopping centre ‘cause I just feel because I can't see properly I just feel so closed in and I worry about where the children are if you know if I and crossing the road, I'm really scared of crossing the road* **Ei: ** *I just generally try to keep it as a normal family, and any difficulties or any erm, problems I have with myself now, such as you know, the difficulty with my vision … . but I will carry on being a mum, I won't sit down and say oh I can't do it my arm hurts, I will go and carry on* **Fi: ** *but it doesn't just affect my life, and my lifestyle, its affected my husbands, it's affected the way we do stuff as a family, you know, cause you have to consider well is, is that going to be too much of a busy day for your mum, or is she going to manage, or, you know, and you have to take into consideration, it's like, well I can't travel too far without getting tired, that makes me very tired* **Gi: ** *if you've had a stroke, my gut feeling, is, it stops you from working, in whatever capacity you had before, now, there are degrees of that don't get me wrong, but, by and large, I think it's lovely that people want to go back to work, but I, I do question whether stroke victims want to go back to work or whether they have to go back to work, when everyone says oh they've gone back to work, cause nearly everybody I know who's a stroke victim's gone back to work. If you talk to them, they don't feel comfortable* **Hi: ** *although they were supportive to me in that I just built on the actual day just again that kind of it's funny how in hindsight you forget how tiring it was everything was tiring they allowed me to build up I think it felt a little bit it was like yeah erm what's that phrase when it's like what you've done where you grow up with you know I think my boss then spoke to I've got another manager and er they got some a bit of a couple of programmes for the computer that would help* **Ii: ** *although I had made significant improvements with a phased return there were all sorts of things going wrong that I didn't understand so I applied for early retirement in 2007. I didn't understand what was going on* **Ji: ** *it was obvious to her from what I was telling her that there was some real severe bullying going on and I suppose as a bloke who's worked and had a senior position for a while you don't believe that that will happen* **Ki: ** *I mean, throughout, I have tried to be positive about this, but in the space of, in a space of 6 months, I lost the house, we lost, we lost, I lost my job, I had the stroke and the heart attack, my mother had a stroke, and we were at risk of losing the house* **Li: ** *in that meantime the erm I stopped driving because I just didn't feel safe anymore* **Mi: ** *there was no suggestion that I had any form of visual problem you know and I was driving erm* **Ni: ** I suppose that was when it hit me that things had drastically changed when they took my driver's licence off me because I was you know up until then I had my own car and I went through about 6 months of just I was an absolute ***** ‘cause I was just so frustrated that my I felt like my freedom had been taken and I was really angry ‘cause I couldn't just jump in the car and fly out here, there and everywhere
Information
**Oi: ** *and it was great, because although I knew I wasn't getting anywhere, but I'd begin to understand what was going on and erm, for someone to sit with me and spend the time to break down what had actually happened and how it had affected my eyesight, and what was going on inside my head, with regards to my vision, not a lot was inside my head, now, but from the vision, from the vision part, it was er, it was quite, it was very interesting, because again, I had now some knowledge and, of what was going on, erm, and I could understand it better, as to why it was happening, erm, and not just left as though it didn't matter and you don't need to know, just get on with it, you know, so erm, I was grateful for that because it put things together, it again, having a stroke's a bit like a jigsaw, trying to put some of your life back together, erm, and it's that information that helps, to, for you to actually move forward with it, erm, although it would be another sort of chapter in your life that you have to adapt to, but to have that part of information, was good, it was good for me* **Pi: ** *I've learnt a lot over the years of having this, erm, and I've, I've met a number of people with vision problems, and it seems that it isn't taken seriously enough, erm, for me, it should, erm, and it's er, neglected, I think, and, and a lot, and I, and I, and I also feel that there's a lot of clinicians out there who really don't understand it* **Qi: ** *it did make a big difference because from then on you know then became registered partly sighted and did help more with erm well just like well it wasn't bus passes at that but you know all sorts of things free* **Ri: ** *lots of bits of paper, lots of leaflets we had a community stroke person on the ward that was pretty useless and er you can't deal with lots and lots of bits of paper, neither can your family you know my wife was sort of clinging on with her fingertips visiting me every day looking after the three lads holding down her job you know and just collecting leaflets* **Si: ** *I got no help erm, from, from the sort of the hospital side of things, and so I took it into erm, well, I'd say my wife erm, took it into her own hands to start sorting something out for herself, and erm, I got in touch with a, erm, the RNIB who were absolutely fantastic, very helpful, erm, and this was my wife that got in touch with them, and they were just so, so helpful erm, and sent lots of information out, erm, how to adapt with things, how to look at things, erm, you, you know, the, and some of the feelings that you go through and, and what's happening to you, so it was, it was, I can't, you know, they were great, absolutely fantastic, erm, and the information that I got, and it was all easy* **Ti: ** *well sometimes you, we used to pick leaflets up, you know the leaflets that so the hospital at least had that information about the Stroke Association* **Ui: ** *I think mine was is it going to change was the main thing, is it going to get worse, will it get better* **Vi: ** *one of the first things I would tell them, I would be as hard as it is and be blatantly honest and tell them that this is it, it's not going to get better, because I really do wish that I knew that so that would probably be one of the first things I told them* **Wi: ** *I think that's it very much felt because he at the beginning as well was finding it hard that we weren't getting information erm and I think a little bit what I've said there is that from early you need to know as much as possible* **Xi: ** *yes, erm, it's, there's no hard and fast answer, right? Erm, but someone (coughs) needs to be able to gauge when the survivor is, has the ability to take in the information that they, that is being received, right? Erm, I would strongly suggest that, as they leave hospital, they're definitely provided with something, not on day of departure*

Typically participants presented to their GP or local optometrist with their visual symptoms. Others presented to A&E if they had associated headache, when migraine was most frequently considered (Table 1B). Stroke was often not a considered diagnosis (Table 1C).

Some reported no urgency in onward referral because of visual symptoms (Table 1D). Even when stroke had been diagnosed there were considerable discrepancies in receiving eye care—noted through the timing of their first referral for visual assessment. Some individuals had immediate referral to ophthalmic services. This was particularly apparent for those with double vision. Many with visual field loss had either a late referral or none at all or were advised to go to their local optometrist (Table 1).

Individuals were often surprised at the results of their eye tests when eventually assessed (Table 1F). They reported how important it was to have an eye test to at least understand what was wrong with their vision (Table 1G). There were clear accounts of treatment being provided successfully for strabismus and eye movement defects. However, lack of treatment was commonplace for visual field loss with many being told nothing could be done for them (Table 1H).

### 3.3 Impact of Symptoms

Participants reported a number of general and visual problems and symptoms following their strokes (Figure 3). In particular, they noted the added impact overall from having visual impairment on top of their other stroke‐induced disabilities, stating that *it makes life more difficult definitely* (Table 1I).

Visual impairment was described by many as a hidden disability that caused considerable problems both when in hospital and when at home (Table 1J). One participant believed he would have been treated differently if clinicians could “see” the visual problem (Table 1K). Conversely, one participant refused to use the term “hidden disability” as he believed more should be done to improve awareness (Table 1L).

Participants described feeling vulnerable because of partial sight (Table 1M). They described the emotional impact to their self through feeling a burden to others, feeling guilt, frustration, and anger (Table 1N). Some of these feelings were at times confused with or attributed to depression but participants were typically clear in stating an opinion on whether they were depressed or not (Table 1O). A common phrase used by participants was “the grieving process” which occurred over, often, lengthy periods with mourning for the person they used to be and difficulty coming to terms with the person they now were and acceptance of their visual impairment (Table 1P).

### 3.4 Self‐made Adaptations

Although some individuals did obtain formal assessment, treatment, and advice at varied time points, many did not. Despite the latter, all participants made self‐determined adaptations to visual impairment, that is, options that they worked out for themselves but not techniques that were shown or taught to them (Table 2).

**Table 2:** Adaptations and impact of visual problems

Visual adaptations	
Magnifiers	Large print
Narrow columns for reading	Increased caution in kitchen
Reading guides/ruler/bands	Kitchen gadgets
Colored overlays on books	Increased lighting
Companion when going out	White stick
Positions things to nonaffected side	Technology—apps.
Sunglasses	De‐clutter
Closing one eye	Compensatory head tilt/turn
Head and eye scanning	
Impact issues	
Loss of confidence	Assistance required when outside
Panic attacks	Unable to pursue hobbies
Fear of falling	Unable to return to work
Startled by sudden appearances from blind side	Fear of dark evenings/nights—worse in winter time
Loss of driving licence	Fear of crowded places
Increases bumps/collisions	Misjudged distances

When reading, participants often switched from paper copies to tablets and e‐readers, used a larger font size and/or used a magnifier (Table 1Q). If reading a paper copy, they would typically use a ruler and a marker at the start/end of lines (Table 1R). In addition to tablets and e‐readers other forms of technology and gadgets were used to facilitate activities. Getting lost easily was described by many participants and satnav or online maps were helpful for this as were applications to help with reading and typing (Table 1S).

Kitchen gadgets were helpful when making food/drinks along with bright labels and fittings to use on appliances (Table 1T). When walking about, a number of participants had obtained white sticks for use outdoors (Table 1U). When indoors, one adaptation was to put a hand out on the impaired side as a guide (Table 1V). Others adopted an active scanning of their environment using head and eye movements to help detect more objects on their partially sighted side (Table 1W). Deliberate position of others, either to the impaired side if out walking, or in the better seeing side if in conversation, were easy and effective adaptations (Table 1X). Those with double vision found that closing one eye or tilting their head provided relief from symptoms. Sunglasses were reported as helpful for glare (Table 1Y).

### 3.5 Daily life

Having visual impairment poststroke impacted on daily life in a number of ways (Table 2). Visual impairment caused the participants to have more collisions because of impaired sight. This was a source of personal danger and physical harm (Table 1Z). There were frequent reports of having the potential to hurt other people which also caused embarrassment to the participants (Table 1Ai).

Loss of confidence was widely reported which participants felt was “under estimated” and “undervalued” leading to altered daily activity (Table 1Bi). Family members often stepped in to help and guide in daily situations to look out for the participant such as when out and about in busy places (Table 1Ci). Those with young families were fearful of not being able to visually monitor their children (Table 1Di). Those with older children would consider masking the full extent of their visual impact (Table 1Ei). They acknowledged that, regardless of the effect the visual impairment had to their own lives, there was an added impact to the lives of their family members (Table 1Fi).

For participants of working age, all considered a return to work if at all possible. However, all faced substantial difficulties (Table 1Gi). Some had positive experiences with the support they received from their employers (Table 1Hi). However, despite supportive work places, many found they could not continue in employment because of limitations related to their vision in addition to general fatigue and lower concentration spans related to their strokes (Table 1Ii). Others had very negative experiences in their attempts to return to work, encountering bullying and harassment (Table 1Ji). This led to worrying concerns regarding the future for themselves and their families (Table 1Ki).

Given the requirement for good vision to be able to drive, it was not surprising that many described driving as an important change in their lives. Some participants took the decision themselves to stop driving, even if they had not received medical advice for this. This was usually because of not feeling safe or not wanting to risk an accident (Table 1Li). Others carried on driving as they had received no warning about visual impairment and were not aware of the extent of their visual deficit, particularly in the early months after stroke: a fact they subsequently acknowledged as being very dangerous at the time (Table 1Mi). Many described the loss of independence on losing their driving licence with some feeling very despondent by this (Table 1Ni).

### 3.6 Information

Many participants described, both positively and negatively, the information and support they received in relation to their stroke and visual impairment. When considering poststroke support, two received a 1‐month community support package after discharge for their general problems, two received information from local vision rehabilitation officers, and one received no support and sought private medical care. Only one received information about visual problems while in hospital. Stroke survivors and their carers felt there was a need for improvement of education to promote knowledge and increased awareness of poststroke visual impairment.

Those that received visual information specific to their type of visual impairment praised this highly as having an important effect on their understanding of what was happening (Table 1Oi). Concurrently they were aware that others in similar situations had not received such information and this was an unmet need (Table 1Pi).

Specific information on partial sight registration was welcomed by many, particularly because of the potential benefits it led to (Table 1Qi). Others received generic information and described this as unhelpful to them (Table 1Ri). Many stated they received no information at all on visual impairment but felt fortunate they were in a position to be able to source information themselves with help from their families (Table 1Si). Some had been able to locate information leaflets left in resource areas on the stroke unit and could trace further support that way (Table 1Ti).

When discussing information that participants would have preferred to receive, they cited help with reading, information on whether the impairments would worsen or, if improved, would return (Table 1Ui). Honesty about the likely prognosis for their visual impairment was important along with information aimed at children and audio versions (Table 1Vi). Very few received support for visual impairment after discharge and, based on their own self‐identified support, cited a number of charities and local groups that should be recommended to stroke survivors: Age concern, Community link, Connect, Different Strokes, Headway, Henshaws, Inspire, the Stroke Association, Speakability. They acknowledged that many other groups would exist depending on location.

The timing of when visual information should be provided was varied. Some considered this should be provided immediately on the stroke units, while others said it should be many weeks later. A number of participants who, through their poststroke voluntary work, acknowledged the varied needs of stroke survivors in the acute stroke stage and, sagely, advocated provision of information immediately so family members could access this, even if the stroke survivors could not yet take this on board (Table 1Wi). They recommended a follow‐up check when approaching discharge to ensure the information was still available to them (Table 1Xi).

## 4 DISCUSSION

The lived experiences and views of stroke survivors with visual impairment have been explored in a series of prospective interviews. The advantage of biographical narrative interviews is that they provide detailed information (Ritchie & Lewis, 2007). This was important to this study in which depth of information was important to obtain insight and understanding to stroke survivor views of visual impairment. However, there are some limitations to consider.

Such interviews may be prone to bias because the research team wishes to highlight certain issues. Bias may also come from the interviewees in that certain individuals may be more likely to participate in interviews, for example, those with negative stories to tell, complaints to make. To counter this, separate independent analysis and coding was undertaken for five transcripts (>10%) for cross‐check capture of codes. Also, when interviewing our participants, we noted that there were as many reporting good medical care as those reporting limits to their medical care. Thus, negativity was balanced by positive participant reports.

A further limitation to interviews is that they may not be generalizable because of small sample sizes. To counter this, we continued interviewing until saturation of data was obtained. We conducted 35 interviews in total but found, on analysis of all interview data, saturation was reached with 28 interviews. Our target sample size of 35 during the design of this study was not intended as “concrete” and, if saturation has not been reached within that target, recruitment of greater numbers would have continued until saturation was reached.

From their interviews, issues were evident with prompt diagnosis in younger stroke survivors, particularly where visual problems occurred. There was a varied age range for our participants. This is reflective of our general stroke population with average stroke onset in the 60–70s but with one quarter of stroke survivors of younger working age. Clearly, there was a discrepancy noted across age groups for prompt diagnosis where visual problems were the main stroke sequelae. Diagnosis was quicker when focal neurological deficits emerged such as hemiparesis and aphasia.

The visual problems experienced by these participants were quite varied but included hemianopic visual field loss, double vision, and nystagmus, facial palsy with dry eye, reading difficulty, glare, blurred vision, and perceptual problems. This is a representative spread of poststroke visual impairment (Rowe & VIS Group, 2009b, 2011, 2013). However, such a heterogeneous group in relation to the varied types of visual impairment must be acknowledged as a potential limitation to the study. It was important to this study to include participants who had experienced varied visual problems across the range of visual categories of reduced central vision, peripheral field loss, eye movement disorders, and visual perceptual defects. Given the previous absence of biographical interviews in this particular stroke/vision population, exploring the lived experiences across the range of visual conditions could highlight clear gaps in service provision alongside the identification of future research questions. It would, however, be beneficial in future qualitative research to provide, where possible, additional information about the participant's clinical profile and functional status.

Vision was affected from the onset of their strokes but attention from medical staff members was always directed to other disabilities first such as mobility, aphasia, and dysphagia. This is understandable but also a problem as a lack of visual information resulted in more bumps and collisions for some participants as they began to mobilize, regardless of participant age. Both issues need to be addressed together so that people are successfully and safely mobilized.

One key inclusion criterion for this study was that participants were at least 1 year post stroke onset. This was on the advice of the study's patient advisory group who believed that stroke survivors would be better able to distinguish vision‐related impact to quality of life versus impacts from other stroke sequelae such as limb weakness or aphasia. The impact of visual impairment to quality of life and daily activities reported by these participants included fear of falling, increased bumps or accidents, missing things, being startled by people suddenly appearing from their affected side, loss of driving licence, a dislike of crowded places, and loss of confidence. These are typical of impacts reported from quality of life questionnaire data (Hepworth & Rowe, 2016). Our participants did not complete any formal quality of life questionnaires as the interviews were, for many, quite lengthy and tiring. Additional questionnaire completion was considered to be too burdensome. Thus, we cannot compare the interview responses of impact to quality of life to formal measures.

Adaptations made to compensate for the visual impairment included having someone with them when out, leaving lights on all the time, and use of a white stick. Other adaptations included use of magnifiers, large print, reading with narrow columns, increased caution, and de‐clutter. There was no consistency for provision of visual assessment and treatment early after stroke and no visual information was provided to any person. These adaptations and reports on assessment, treatment and provision of information, were consistent across all age groups and across the different types of visual impairment. Poor care provision with resultant unmet need for stroke survivors who experience visual impairment has been previously documented (Rowe et al., 2015). Clear recommendations exist for the delivery of appropriate service provision (Rowe et al., 2016).

Misinformation such as “hemianopia will recover over a few years” or “hemianopia due to migraine” angered participants. Postdischarge support was also lacking which is disappointing given that many local services can provide considerable support and assistance.

## 5 CONCLUSIONS

This study provides information about the impact of poststroke visual impairment to quality of life through stroke survivors’ accounts of their lived experiences. Improved knowledge and awareness of the visual problems that can occur due to stroke was perceived by many as being an important future change to make. Participants had not been aware that vision could be affected by a stroke and made reference to clinicians being inattentive to the possible presence of visual impairment. This potentially served to delay diagnosis of stroke in some cases. Furthermore, stroke survivors need early vision assessment after onset of stroke so that this information is available to the stroke team to influence their care provision. Early provision of visual information is reported as beneficial by stroke survivors as is postdischarge information about local support services.

## CONFLICT OF INTEREST

The author reports no conflict of interest for this study.
